# A three minute- screening tool for temporomandibular joint involvement in children with juvenile idiopathic arthritis

**DOI:** 10.1186/1546-0096-12-S1-P180

**Published:** 2014-09-17

**Authors:** Gabriella Giancane, Michiel Hendrik Steenks, Harriette Willemijn van Bruggen, Rob de Leeuw, Nico Wulffraat

**Affiliations:** 1 Department of Pediatric Immunology, UMC, Utrecht, Netherlands; 2 Department of Oral-Maxillofacial Surgery, Prosthodontics and Special Dental Care, UMC, Utrecht, Netherlands; 3 Department of Statistics, Julius center, UMC, Utrecht, Netherlands

## Introduction

The temporomandibular joint (TMJ) is a possible localization of arthritis in patients with Juvenile Idiopathic Arthritis (JIA), with an estimated prevalence around 50%. Despite the decreasing involvement in the last years, maybe due to the current treatment strategies, TMJ remains one of the most clinically under recognized affected joints in JIA patients. The clinical examination of TMJ is often unreliable, even for experienced rheumatologists, leading to the need of more specific diagnostic tools.

## Objectives

Aim of our study was to test a diagnostic score specific for TMJ, capable to shortly detect arthritis in the joint, as assessed by rheumatologist and dentist, the two specialists mainly involved in TMJ examination.

## Methods

78 consecutive patients (5-17 years of age), all affected by JIA, according to the ILAR classification, were selected in the outpatient clinic of our department. 76 gave informed consent and were examined between December 2013 and January 2014 (Table 1). Those with dental and facial disorders were excluded. Four rheumatologists were trained in TMJ examination by a dentist. On the day of consultation, the patient was first examined by the rheumatologist and then by the dentist, considered the gold standard examiner and blind to the findings of the rheumatologist. Both specialists were asked to use separately the score for the TMJ evaluation, composed by 11 items: five concerning patient history and six based on physical examination. Each item was scored as 1, when present, with a total score ranging from 0 (no TMJ involvement) to 11 (maximum severity of TMJ involvement). Juvenile Idiopathic Disease Activity Score (JADAS) was also calculated for each patient and correlated with the TMJ score. The internal consistency of the tool and agreement among specialists were determined through kappa statistics and pearson coefficient.

## Results

74 JIA patients (M/F 2.4:1), with a mean age of 12 years, were examined. The prevalence of TMJ involvement was 4-11%. The TMJ score required around three minutes to be used. The internal consistency of the tool was 0.78. Deleting the morphological items ‘asymmetry’ and ‘mandibular retrognathism’, with low agreement, the coefficient increased up to 0.85. For the history related items, the value ranged between 0.46 and 0.87; for the examination related items between 0.25 and 0.73. The kappa value for the total score, between the rheumatologist and the dentist, was 0.46. The tool seemed to correlate with JADAS, improving its value in indicating patients to refer to the specialist.

## Conclusion

The TMJ score used in our study showed to be an easy tool, not time-consuming for the rheumatologist, to evaluate the TMJ in JIA patients. The internal consistency of the score was good. The low agreement between specialists highlights the need of adopting further measures, such as more specific training and attention on the examination of TMJ, in order to improve the skills of the rheumatologist in examining this joint. Our score does not aim in giving a validated tool, but it represents a practical instrument for the rheumatologist to support the diagnosis of TMJ arthritis and the possible referral to a gnathologist.

## Disclosure of interest

None declared.

